# Encoding Justice with Data: Environmental Justice Screening Tools and the Limits of Quantification

**DOI:** 10.1007/s40572-026-00543-9

**Published:** 2026-05-30

**Authors:** Misbath Daouda, Carlos F. Gould

**Affiliations:** 1 Division of Environmental Health Sciences, School of Public Health, University of California, Berkeley, Berkeley, CA 94720 USA; 2 School of Public Health, University of California San Diego, La Jolla, San Diego, CA USA

**Keywords:** Environmental justice, Environmental exposures, Data science, Government, Resource allocation

## Abstract

**Purpose of Review:**

This review examines government-sponsored environmental justice indices used in the United States, focusing on how their design choices operationalize justice. Rather than evaluating index outputs, we analyze their structural features, aggregation strategies, and underlying assumptions, and assess how they are used in peer-reviewed research between 2020 and 2025.

**Recent Findings:**

Across jurisdictions, indices show strong structural convergence, typically including exposure and vulnerability domains. Additive and multiplicative aggregation approaches encode different assumptions about how burdens interact, affecting community rankings and designations. Despite growing policy influence, indices are infrequently used as primary analytical tools in research. Procedural and recognitional dimensions remain weakly represented, and index construction is rarely examined explicitly.

**Summary:**

Environmental justice indices function not only as measurement tools but as governance mechanisms shaping eligibility and resource allocation. While effective for distributive screening, they represent partial definitions of justice and should be interpreted with caution within broader democratic and community-based frameworks.

**Supplementary Information:**

The online version contains supplementary material available at 10.1007/s40572-026-00543-9.

## Introduction

Environmental justice has become data-driven [1]. Government agencies and researchers increasingly use screening and mapping tools (e.g., EPA’s EJSCREEN, the federal Climate and Economic Justice Screening Tool [CEJST], the Environmental Justice Index [EJI]) to combine modeled environmental burdens with sociodemographic, health, and infrastructure data, and identify communities facing disproportionate environmental risks. Built on contemporary data science and using applied statistical modeling, computational infrastructure, and deliberate choices about which variables to include, these tools now inform permitting, enforcement, and the allocation of climate and infrastructure investments. For researchers and practitioners, environmental justice indices can be appealing because they offer a way to synthesize complex, multidimensional information into a single and scalable measure that can be operationalized for decision-making. They are also used in scholarship that seeks to document disparities in exposure and risk [2–4].

The rise of environmental justice screening tools coincides with broader changes in environmental governance; agencies face limited enforcement capacity, rely more on decentralized decision-making, and seek standardized metrics to support administrative processes. Consequently, composite indices have emerged as a way to translate broad ideas about environmental justice into concrete policy decisions, particularly where agencies must allocate limited resources using consistent criteria [5–7]. But, they also reshape the way justice claims are made and understood. Historically presented through narrative, legal argument, and community testimony, environmental justice is increasingly codified with rankings, thresholds, and eligibility criteria [7, 8]. Environmental justice indices are therefore not just measurement tools: because each index embeds assumptions about what counts as injustice and who should receive priority for intervention, each one functions as a governance mechanism that embodies a specific theory of justice.

Environmental justice scholarship provides the frame for interpreting what such indices can and cannot represent [9–11]. Much of the quantitative environmental justice literature has centered distributive justice: how environmental harms and benefits are allocated across populations and places [12–16]. Yet environmental justice frameworks also emphasize procedural justice (who participates in decision-making and with what power), recognitional justice (whether institutions acknowledge community histories, identities, and lived realities), and self-determination (whether communities define priorities and evaluate success) [17–19]. Drawing on foundational definitions [17] and contemporary applications [20–22] of environmental justice, we focus on its distributional, procedural, and recognitional dimensions. However, we recognize that environmental justice increasingly incorporates an intergenerational perspective, emphasizing that environmental decisions made today shape risks, opportunities, and capabilities for future generations. The field has also stressed cumulative impacts, understood as the overlap of multiple stressors, often motivating methods and practices that extend beyond administrative datasets and resist single scores [2, 23–25]. These include right-to-know regimes, community monitoring, participatory governance, and other mechanisms that expand public accountability [26–30].

Because procedural, recognitional, and community-defined dimensions of justice are harder to standardize in national administrative data, screening tools tend to default to what can be measured and compared at scale. As a result, core questions about environmental justice are increasingly answered through data-scientific design choices. Decisions that appear technical [31]—which factors to include (feature selection), how to represent them (transformation and normalization), which carry the most weight, and how they get combined—embed judgments about what is (or is not) injustice. For example, one index might weight pollution concentrations over housing conditions, or current exposures over historical disinvestment, leading to different communities being identified as the most burdened. In this way, these choices do not merely summarize inequality; they shape which communities are designated as burdened, disadvantaged, or eligible for intervention [32].

Prior research has examined environmental justice indices primarily as technical tools for identifying disparities or prioritizing regulatory action, including critiques of indicator selection, correlations among inputs, or alignment with health outcomes [33]. Related scholarship has demonstrated that metrics, classifications, and algorithms actively shape how social problems are defined and governed [6, 34, 35]. For example, after *U.S. News & World Report* rankings became influential, law schools changed admissions criteria, enrollment strategies, and resource allocation in order to improve their rank, gradually internalizing the metric as a definition of quality. In doing so, the ranking system came to define what counted as institutional success, structured day-to-day decision-making within law schools, and contributed to producing the very hierarchies it aimed to measure [36]. Less attention has been paid to environmental justice screening tools at the intersection of these literatures: as instruments that operationalize data-scientific choices to define justice in practice.

In this review, we examine government-sponsored environmental justice indices used in the United States, focusing on their construction rather than their results. We describe common structural elements, aggregation strategies, and design choices across tools, and situate these choices within broader environmental justice frameworks. We argue that contemporary indices operationalize justice primarily as relative distributive burden, while procedural, recognitional, and community-defined dimensions remain weakly represented. Despite this, these indices are increasingly used as administrative decision rules in high-stakes policy settings, even though their underlying assumptions and construction receive limited attention in the environmental justice literature. This gap underscores the need to more clearly articulate and evaluate the data-scientific foundations of these tools, rather than treating them as objective measures of injustice. Our review of peer-reviewed literature published between 2020 and 2025 similarly suggests that index construction is rarely examined directly, even as these tools become more central to policy and governance.

## Methods

We conducted a review of government-sponsored environmental justice indices published between 2020 and 2025 and currently used in the United States to inform policy, regulatory decision-making, and resource allocation. Our objective was not to evaluate the empirical validity of the indices’ outputs, but to examine how these tools are constructed and how their design choices operationalize particular conceptions of environmental justice. This article does not contain any studies with human or animal subjects performed by any of the authors.

We defined an environmental justice index as a tool that produces either a composite score or a composite designation from multiple indicators, and explicitly intended for ranking, prioritization, or eligibility determination along environmental justice dimensions. To be included, a tool had to meet three criteria: (1) it had to be explicitly described as a composite index (as opposed to a visualization of independent data layers); (2) it had to be developed, maintained, sponsored, or formally adopted by a government agency at the federal, state, or local level; and (3) it had to have an official public-facing webpage or technical documentation describing its construction, underlying indicators, and scoring approach.

Indices were identified by first drawing on prior reviews of environmental justice indices (e.g., Joseph L, 2025), and then supplemented through targeted keyword searches (e.g., “environmental justice index,” “environmental justice screening tool”) of government agency websites and the academic and policy literature. Indices were then screened against the definition described above. Additionally, indices that appeared to be in governmental use but lacked sufficient public documentation at the time of review were catalogued for completeness but excluded from detailed methodological comparison.

For each eligible index, we extracted information on its stated purpose, geographic scope, unit of analysis, and version history. We then reviewed the corresponding technical documentation to characterize how each index was constructed, focusing on indicator categories, data transformations, aggregation methods, and weighting choices. Where documentation permitted, we recorded how indicators were normalized (e.g., percentiles or deciles), how intermediate scores were defined, and how final composite scores or classifications were generated.

**Fig. 1:**
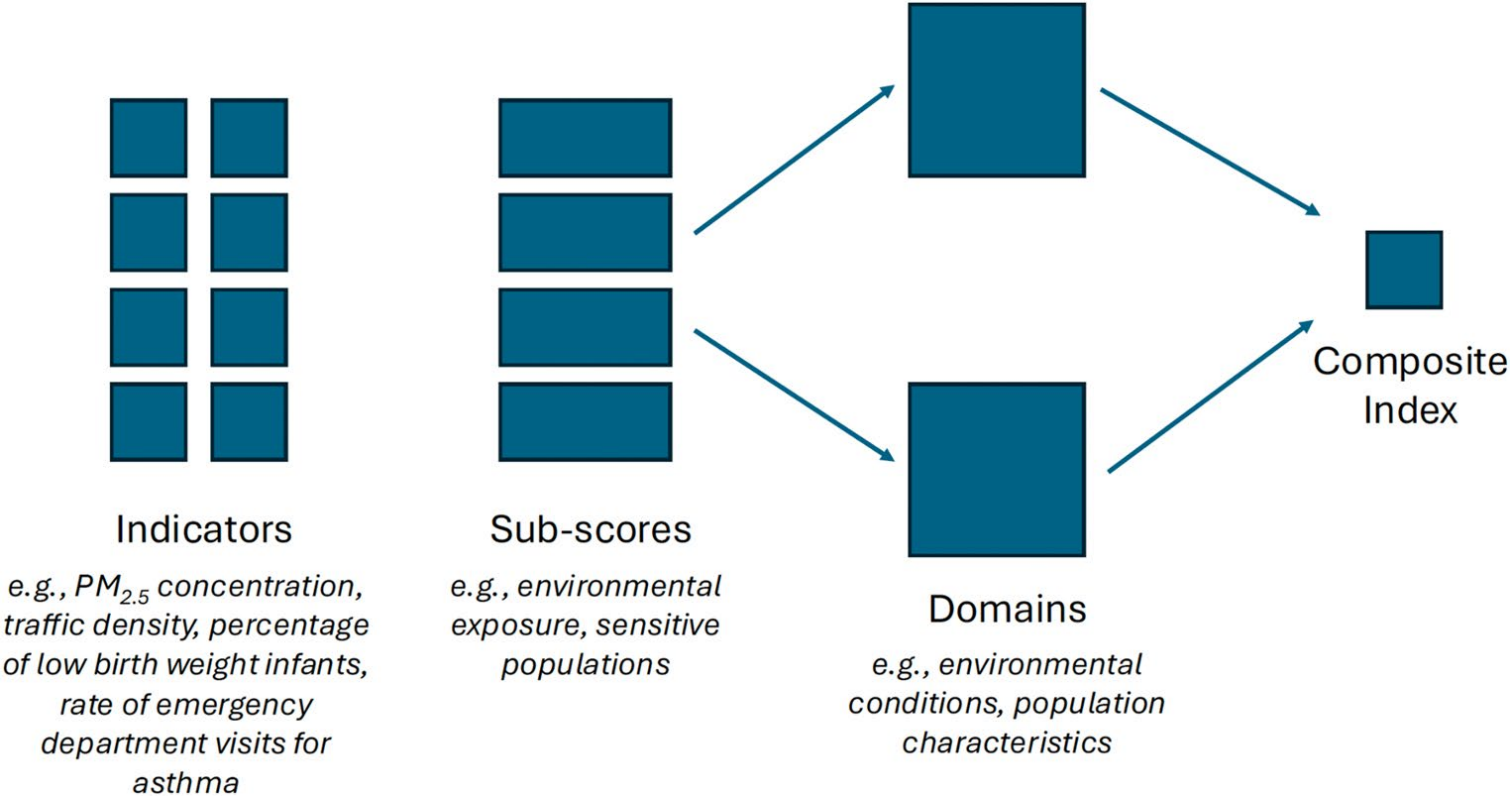
Illustration of the nomenclature adopted to describe the structure of identified indices. Figure created by the authors

To facilitate comparison across tools, we adopted a consistent terminology reflecting the hierarchical structure common to most indices (Fig. 1). At the lowest level are *indicators*, representing measured environmental, social, or health-related variables. Indicators are typically aggregated into intermediate groupings, referred to here as *sub-scores*, and then combined into higher-level *domains*, most commonly an environmental burden domain and a population vulnerability domain. Final outputs take the form of continuous *composite scores* derived through additive or multiplicative logic.

In some cases, thresholds are applied to composite scores to generate categorical designations, such as “disadvantaged community,” which can be used to define eligibility for targeted investments. Where available, we documented these thresholds.

To assess how environmental justice indices are used in scientific research, we conducted a targeted review of the peer-reviewed literature between 2020 and 2025. The search was conducted on Google Scholar on February 2, 2026 and combined three conceptual elements: first, we specified our objects of interest using generic search terms such as “environmental justice index” and “environmental justice screening tool” as well as the name of each of the specific tools identified in the policy review (e.g., EJScreen, CalEnviroScreen); second, we included key terms to identify cases where the index was used in quantitative analyses (e.g., “regression”, “modeling”, “effect modification”, “risk assessment”); and third, we included exposure and outcome related terms relevant to environmental epidemiology (e.g., “health”, “asthma”, “exposure”, “PM2.5”, “emissions”). Our aim was not to produce an exhaustive bibliometric analysis, but to identify patterns in how these tools function within research designs. We identified studies in which government-sponsored environmental justice indices were used as central analytical variables rather than as illustrative background maps. We classified these studies according to the role the index played in the analysis, including descriptive distribution of hazards and health outcomes, heterogeneity analyses in exposure-response modeling, policy targeting and distribution of benefits, and as sources of data on social determinants of health. For each study, we recorded how the index was operationalized and which dimensions of justice were primarily engaged.

## Results

**Fig. 2:**
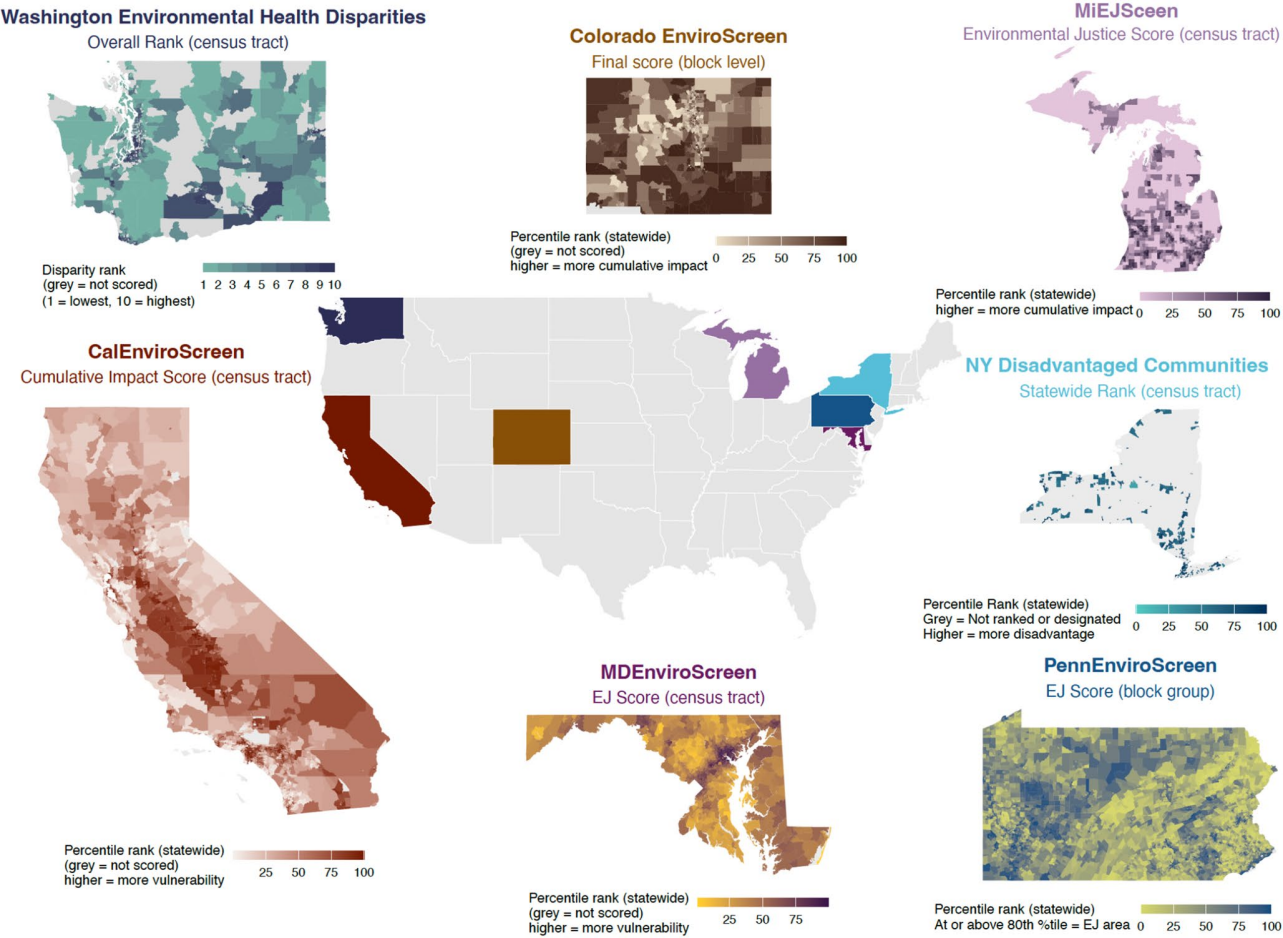
State-level environmental justice screening indices and outputs

Table 1 summarizes government-sponsored environmental justice screening tools currently in use in the United States. At the federal level, only two of the four identified indices—CEJST and EJI—are publicly documented and accessible, both operating nationwide at the census tract scale. The other tools are state- or locality-specific, with wide variation in geographic coverage, unit of analysis, stated purpose, and documentation quality. After excluding indices that did not make their underlying technical documentation available, the seven states represented in this study are California, Colorado, Maryland, Michigan, New York, Pennsylvania, and Washington (Fig. 2). The spatial unit most commonly used across these state-specific indices is the census tract with only two indices using census block groups.

**Table 1:** Overview of government-sponsored environmental justice indices

Tool	Government Sponsor	Stated Purpose	Coverage	Spatial Unit	Latest version*
EJSCREEN	United States Environmental Protection Agency	*Documentation unavailable*	Nationwide	Census block	2024
Climate and Economic Justice Screening Tool (v2)	White House Council on Environmental Quality	“to identify disadvantaged communities facing particular climate, environmental, and economic burdens.”	Nationwide	Census tract	2024
Environmental Justice Index	Center for Disease Control - Agency for Toxic Substances and Disease Registry	“the first place-based nationwide index designed to address cumulative impacts through the lens of EJ and health equity.”	Nationwide	Census tract	2024
Energy Justice Mapping Tool	United States Department of Energy	*Documentation unavailable*	Nationwide	Census tract	2024
CalEnviroScreen (v4)	California Environmental Protection Agency - California Office of Environmental Health Hazard Assessment	To address the cumulative effects of pollution burden, socioeconomic stressors, and health conditions, and to identify which communities might be in need of particular policy, investment, or programmatic interventions	California	Census tract	2021
Colorado EnviroScreen	Colorado Department of Public Health and Environment	“To support environmental justice activities among government entities, industry, community organizations, and residents in the state of Colorado”	Colorado	Census block group	2024
MDEnviroScreen	Maryland Department of the Environment	“To help identify communities disproportionately burdened by multiple sources of pollution and with population characteristics that make them more sensitive to pollution”	Maryland	Census tract	2025
Massachusetts EJ Viewer	Massachusetts Executive Office of Energy and Environmental Affairs	*Documentation unavailable*	Massachusetts	Census block group	2022
MiEJScreen	Michigan Department of Environment, Great Lakes, and Energy	“To identify communities most affected by cumulative environmental health impacts”	Michigan	Census tract	2022
New Jersey EJMAP	New Jersey Department of Environmental Protection	To determine if an area’s environmental and public health stressors are higher than its geographic point of comparison	New Jersey	Census tract	2025
New York Disadvantaged Communities Map	New York State Energy Research and Development Authority	To identify and consider disadvantaged communities (DAC) in regulatory actions and implementation of the Climate Act	New York	Census tract	2023
PennEnviroScreen	Pennsylvania Department of Environmental Protection	The primary purpose is to assist the Pennsylvania Department of Environmental Protection staff in implementing an Environmental Justice Policy	Pennsylvania	Census block group	2023
Virginia EJScreen+	Virginia Department of Environmental Quality	*Documentation unavailable*	Virginia	Census tract	
Washington Environmental Health Disparities Map	Washington State Department of Health	“To inform state environmental policy, budgeting priorities and regulation enforcement to reduce health inequities across communities”	Washington	Census tract	2022
Chicago Environmental Justice Index	City of Chicago	To produce “a relative, rather than absolute, measure of cumulative impact”	Chicago	Census tract	2023
Los Angeles County Environmental Justice Screening Method	Los Angeles Department of Regional Planning	*Documentation unavailable*	Los Angeles County	Census tract	2023

*The latest available version was considered the first version unless specified

Maps show the primary environmental justice index outputs produced by selected U.S. states, displayed at the index’s native resolution (often census tract). Each map visualizes the final score, rank, or designation used by the state to identify communities facing elevated cumulative environmental and social burdens. Darker shading indicates higher relative burden or disadvantage within each state based on the index’s defined scale. Grey areas indicate census tracts that are not scored or designated, including tracts outside the index’s coverage or excluded due to data or eligibility criteria. Because these indices differ in their underlying indicators, aggregation methods, and policy purposes, values are not comparable across states. Tables 1 and 2 provide detailed documentation of each tool’s stated purpose, indicator categories, aggregation methods, and designation criteria. Figure created by the authors.

**Table 2:** Description of index construction

Tool	Categories of indicators	Indicator format	Temporal range of contemporary indicators	Formula	Aggregation method	Weighing choices	Designation
Climate and Economic Justice Screening Tool	Environmental, climate, or other burdens, socioeconomic burdens	Percentile rank	2014–2022	(1) at or above the threshold for one or more environmental, climate, or other burdens; and (2) at or above the threshold for an associated socioeconomic burden.	N/A	Equal weight	A census tract is identified as disadvantaged if it is (1) at or above the threshold for one or more environmental, climate, or other burdens; and (2) at or above the threshold for an associated socioeconomic burden. In addition, a census tract that is completely surrounded by disadvantaged communities that meets the burden thresholds, and is at or above the 50th percentile for low income, is also considered disadvantaged.
Environmental Justice Index	Social vulnerability, environmental burden, health vulnerability	Percentile rank	2018–2024	Score = Social vulnerability module [percentile ranked sum of social vulnerability indicators] + Environmental burden module [percentile ranked sum of environmental burden indicators] + Health vulnerability module [ranking calculated from health vulnerability flags]	Additive	Equal weight	
CalEnviroScreen	Exposures, environmental effects, sensitive populations, socioeconomic factors	Percentile rank	2015–2021	Score = Scaled pollution burden [average of exposures and environmental effects] x Population characteristics [average of sensitive populations and socioeconomic factors]	Multiplicative	Environmental effects score is weighed half as much as the exposures score	
Colorado EnviroScreen	Sensitive populations, demographics, environmental exposures, environmental effects, climate vulnerability	Percentile rank	2015–2024	Score = Scaled health and social factors score [(average percentile of sensitive populations + average percentile of demographics)/2] x Scaled pollution and climate burden score [(average percentile of environmental exposures + 0.5 * average percentile of environmental effects + 0.5 * average percentile of climate vulnerability)/2]	Multiplicative	Environmental effects and climate vulnerability scores are weighed half as much as the environmental exposures score	
MDEnviroScreen (EJ score)*	Pollution burden exposure, pollution burden environmental effects, sensitive populations	Percentile rank (with 75th percentile threshold set for pollution burden exposure)	2019–2024	Score = ((Pollution Burden Exposure Percentile) + (Pollution Burden Environmental Effects Percentile) + (Sensitive Population Percentile)) / 3	Additive	Equal weight	
MiEJScreen	Environmental exposures, environmental effects, sensitive populations, socioeconomic factors	Percentile rank	2014–2020	Score = Environmental conditions score [(average percentile of environmental exposures + 0.5 x average percentile of environmental effects)/1.5] x Population characteristics score [(average percentile of sensitive populations + average percentile socioeconomic factors)/2]	Multiplicative	Environmental effects score is weighed half as much as the exposures score	
New York Disadvantaged Communities Map	Potential pollution exposures, land use associated with historical discrimination or disinvestment, potential climate change risks, income, race/ethnicity, health impacts and burdens, housing, energy, communications	Percentile rank	2010–2021	Score = Environmental burdens and climate change risks [(weighted average of percentile ranks of potential pollution exposure indicators + weighted average of percentile ranks of land use indicators + 2*weighted average of percentile ranks of climate risk indicators) / 4] + Population characteristics and health vulnerabilities [(weighted average of income indicators + weighted average of race/ethnicity indicators + weighted average of health indicators + weighted average of housing indicators)/4]	Additive	Climate risks are given double weight within the environmental burdens and climate change risks component to match the combined weight of pollution exposures + land use	35% of tracts are designated as DAC based on statewide and regional thresholds for combined score percentile ranks
PennEnviroScreen	Environmental exposures, environmental effects, sensitive populations, socioeconomic factors	Percentile rank	Not stated	Score = Scaled pollution burden score [(average percentile of environmental exposures + 0.5 x average percentile of environmental effects)/1.5] x Scaled population characteristics score [(average percentile of sensitive populations + average percentile of socioeconomic factors)/2]	Multiplicative	Environmental effects score is weighed half as much as the exposures score	Each block group is determined to be an EJ area if its final score > = 80th percentile
Washington Environmental Health Disparities Map	Environmental exposures, environmental effects, sensitive populations, socioeconomic factors	Percentile rank	2014–2021	Score = Pollution burden score [(Average decile rank of environmental exposures indicators +(0.5 × average decile rank of environmental effects indicators)) / 2] x Population characteristics score [(Average decile rank of sensitive population indicators + average decile rank of socioeconomic factors indicators) / 2]	Multiplicative	Environmental effects score is weighed half as much as the exposures score	
Chicago Environmental Justice Index	Environmental exposures, environmental conditions, sensitive populations, socioeconomic factors	Percentile rank	2015–2023	Score = Scaled pollution burden score [(average percentile of environmental exposures + 0.5 x average percentile of environmental conditions)/1.5] x Scaled population characteristics score [(average percentile of sensitive populations + average percentile socioeconomic factors)/2]	Multiplicative	Environmental Conditions is weighted half as much as the Environmental Exposures because the Condition indicators represent risk of exposure, whereas Exposure indicator represent actual measured exposures.	Any census tract with a Chicago EJ Index Score of 75 or greater, or whose Chicago EJ Index score is 70 or greater and contiguous with another census tract with a Chicago EJ Index Score of 75 or greater is designated as an EJ Neighborhood.

*Information extracted for the EJ Score component of MDEnviroScreen; does not include the climate vulnerability score

A small number of jurisdictions, most notably California (Box 1), have developed mature tools with multiple released versions and detailed technical documentation. In contrast, many states rely on indices that are newly launched, under revision, or inconsistently documented. Several tools identified during the review were temporarily unavailable or lacked accessible methodological documentation, indicating that environmental justice screening infrastructure remains fragmented, institutionally uneven, and vulnerable to changes in political leadership.

**Table:** Box 1. Procedural decisions in CalEnviroScreen

1. Stakeholder engagement and public comment
CalEnviroScreen was developed and revised through iterative public processes led by OEHHA, including workshops, advisory groups, and formal public comment periods. Community organizations, researchers, and agencies were able to propose indicators, critique methods, and comment on draft versions. These processes influenced decisions such as which indicators were included, how domains were structured, and how scores were interpreted.
2. Transparency and documentation
OEHHA publishes detailed technical documentation for each version (including data sources, transformations, weighting, and aggregation), along with responses to public comments. Versioning (e.g., 1.0 → 4.0) and archived materials allow users to trace how methodological choices have evolved over time. This supports reproducibility and enables external scrutiny.
3. Indicator selection criteria
Indicators are selected based on a combination of conceptual relevance (alignment with cumulative impacts), data availability at the census tract level, statewide coverage, and statistical properties (e.g., variability, interpretability). These criteria are procedural decisions that determine what “counts” as environmental burden or vulnerability within the index.
4. Weighting and aggregation rules
The decision to use a multiplicative structure (pollution burden × population characteristics), and to downweight “environmental effects” relative to direct exposures, reflects a procedural judgment about how different components should interact. These choices were informed by both technical considerations and stakeholder input.
5. Geographic resolution and comparability
The choice to operate at the census tract level and to use statewide percentile rankings reflects procedural priorities around comparability, administrative usability, and alignment with policy implementation (e.g., SB 535, GGRF allocation).
6. Policy linkage and thresholds
CalEnviroScreen is explicitly designed to support regulatory and funding decisions. Procedural decisions include how scores are translated into designations (e.g., top 25% most burdened tracts) that determine eligibility for programs. These thresholds are not purely technical—they are governance choices with distributional consequences.

Despite this institutional heterogeneity, the stated purposes of these tools are highly convergent. Nearly all are framed as mechanisms to identify communities facing disproportionate environmental burdens or cumulative impacts, often explicitly to support regulatory implementation, investment prioritization, or compliance with environmental justice mandates.

### Structural Features of Indices

Indices exhibit consistent internal structure (Table 2). Nearly all indices organize indicators into two primary domains: an exposure or pollution burden domain and a vulnerability or population characteristics domain. Exposure-related domains typically distinguish between indicators intended to capture direct environmental exposures (e.g., modeled air pollution concentrations, proximity-based exposure metrics) and those representing environmental effects or conditions (e.g., traffic density, land use characteristics). Vulnerability-related domains most often combine indicators of sensitive populations (e.g., age, health status) with socioeconomic factors (e.g., income, education, housing). This two-domain structure was present across all federal, state, and local tools.

All reviewed indices rely on relative ranking of indicators, most commonly through percentile ranks or deciles. Absolute exposure levels or health metrics are not included in final scores. Instead, percentile rank values indicate where a community falls compared with all others without capturing the magnitude of differences between indicator values. This approach reduces sensitivity to skewed distributions and outliers and yields scores that reflect relative standing within a given geography rather than absolute risk or burden. This facilitates comparisons across heterogeneous indicators and regions but also ties interpretation to rank order of the largest chosen spatial unit rather than health-based standards or regulatory thresholds.

### Aggregation Strategies and Implicit Assumptions

**Fig. 3:**
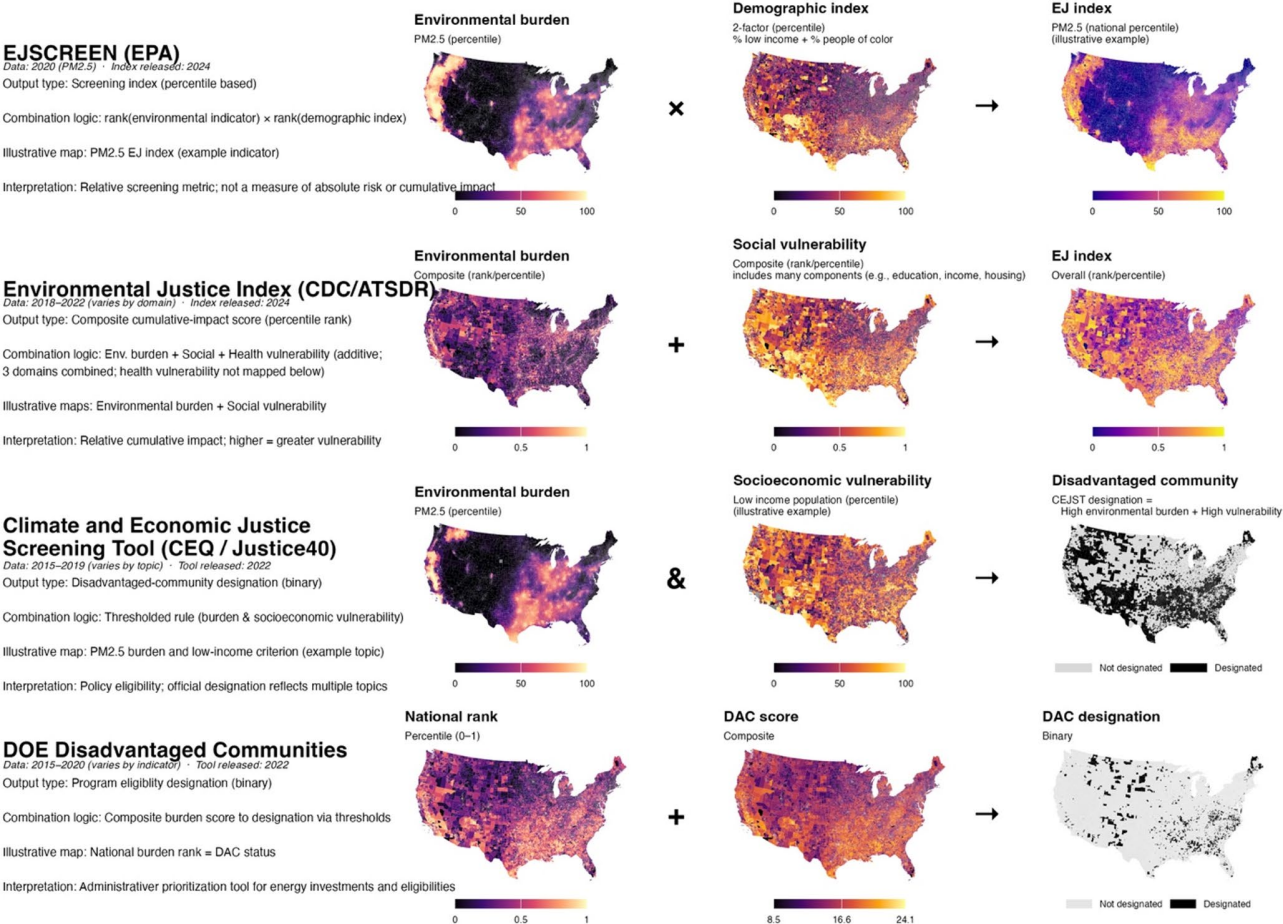
Data-scientific structure of selected U.S. environmental justice screening tools

Indices differ most clearly in how they aggregate ranked indicators into composite scores. Two dominant approaches are used: additive aggregation and multiplicative aggregation. These differences in aggregation logic can substantially influence the relative ranking of communities and, in turn, which communities are ultimately identified as disadvantaged (Fig. 3).

Each row illustrates the internal logic of a widely used government-sponsored environmental justice tool, showing how environmental burden and population vulnerability indicators are transformed, combined, and interpreted. Columns depict (from left to right) the index architecture, a representative environmental burden input, a representative vulnerability input, and the resulting composite score or designation. All tools rely on relative ranking of indicators (typically percentiles or deciles) rather than absolute exposure or risk levels, but differ in how ranked inputs are aggregated. Some indices combine burden and vulnerability additively to produce continuous composite scores, whereas others use multiplicative or threshold-based logic to emphasize the co-occurrence of high burden and high vulnerability or to generate binary designations used for eligibility and prioritization. For tools that support multiple indicators or domains, maps show one illustrative example (e.g., PM2.5) to demonstrate index structure; full implementations incorporate additional inputs as specified in technical documentation. The EJI draws on three vulnerability domains (Environmental Burden, Social Vulnerability, and Health Vulnerability); the figure shows Environmental Burden and Social Vulnerability to maintain a consistent two-input layout across all tools. Together, the figure highlights how data-science design choices—feature selection, normalization, aggregation, and thresholding—encode particular operational definitions of environmental justice. Each index reflects its most recent publicly available release at time of analysis; index vintages vary by tool (CEJST and DOE DAC: 2022; EJSCREEN and EJI: 2024). Figure created by the authors.

**Fig. 4:**
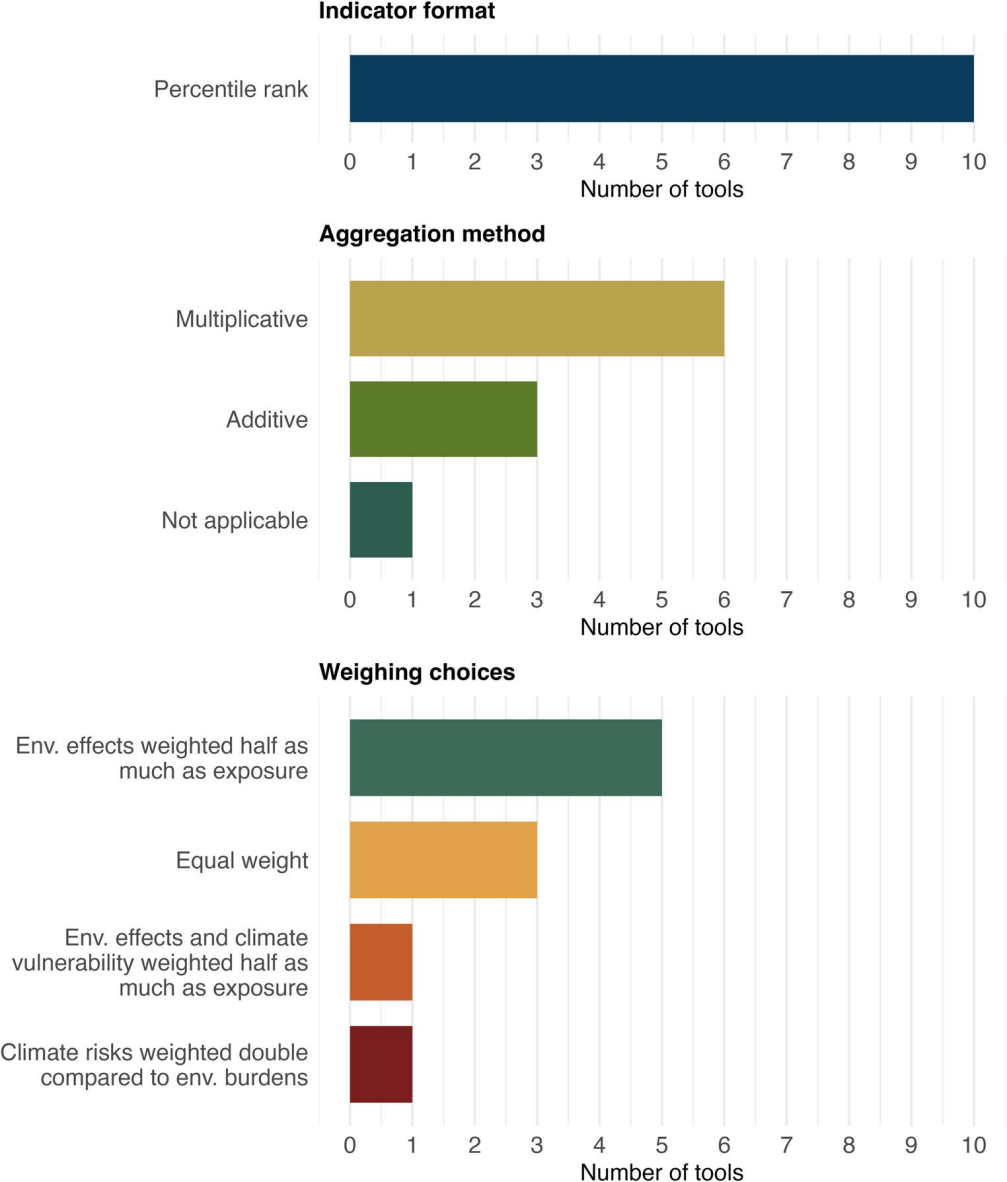
Design choices across identified environmental justice indices. This figure presents counts of environmental justice tools identified by indicator format, aggregation method, and weighting choices. Figure created by the authors

Three out of the ten indices identified sum or average standardized domain or sub-score percentiles (Fig. 4); these additive indices implicitly treat each component as contributing independently and proportionately to overall disadvantage. EJSCREEN represents the simplest application of this approach, relying on percentile-ranked indicators that are interpreted relative to thresholds rather than formally aggregated into a single composite score. EJI adopts an explicit additive structure, summing percentile-ranked social vulnerability, environmental burden, and health vulnerability domain scores with equal weight. MDEnviroScreen similarly averages percentiles across its exposure, environmental effects, and sensitive population domains. Although the number and type of domains vary, these additive approaches share a linear assumption about how burdens and vulnerabilities combine within the composite score.

In contrast, a second group of indices (six out of the ten indices identified) employs multiplicative aggregation to link pollution burden and population vulnerability domains. Tools such as CalEnviroScreen, Colorado EnviroScreen, MiEJScreen, PennEnviroScreen, Washington’s Environmental Health Disparities Map, and the Chicago Environmental Justice Index calculate domain-level sub-scores for environmental exposures and environmental effects, combine them into a pollution burden domain, and then multiply this domain by a population characteristics domain composed of sensitive population and socioeconomic sub-scores. This structure ties the composite score to the co-occurrence of environmental stressors and population vulnerability, producing higher scores in communities where both are high. Within these multiplicative frameworks, most indices give less weight to environmental effects or condition sub-scores than to direct exposure sub-scores, reflecting the assumption that measured or modeled exposures are more directly linked to harm than broader environmental conditions.

Several indices introduce modifications that reflect jurisdiction-specific priorities. Colorado EnviroScreen incorporates climate vulnerability as an additional sub-score within the pollution burden domain. New York’s Disadvantaged Communities Map extends the domain structure to include land use associated with historical discrimination or disinvestment and assigns double weight to climate risk indicators within the environmental burdens and climate change risks domain. Washington’s Environmental Health Disparities Map adopts decile rankings rather than percentiles for indicator transformation, but otherwise retains a domain and sub-score structure closely aligned with the original CalEnviroScreen multiplicative model.

CEJST is the only tool in this set that does not use either additive or multiplicative aggregation to generate a composite score. Instead, CEJST employs a threshold-based classification framework that identifies disadvantaged communities based on whether census tracts meet specified cutoffs across multiple burden categories. Tracts are designated as disadvantaged if they (1) exceed a high national percentile threshold for at least one environmental, climate, or other burden and (2) meet an associated socioeconomic criterion, most commonly low income. In practice, this means that CEJST identifies tracts experiencing co-occurring forms of disadvantage across domains but does not rank places by the cumulative magnitude of burden.

### How Environmental Justice Indices are Used in Scientific Research

Despite their growing prominence in policy and governance, government-sponsored environmental justice indices are used infrequently as primary analytical tools in the peer-reviewed literature. A substantial body of research on environmental inequality instead relies on individual sociodemographic variables, neighborhood deprivation indices, or study-specific cumulative exposure measures (e.g. [14, 16, 37–45]), . In practice, an emerging role for environmental justice indices is as a curated source of environmental and social indicators that researchers use for other purposes.

Here, and in Table 3, we summarize some recurring roles for environmental justice indices in scientific research.

**Table 3:** How environmental justice indices are used in research

Use case	What the index is used for	Primary justice dimension(s) engaged	Refs.	Illustrative examples
Hazard distribution mapping	Outcome is exposure or hazard, and an index is used to stratify or order geographic units to characterize inequalities in exposure burdens	Distributive justice (who bears exposure burdens)	[46–52]	Wen et al. (2024), *Environmental Science & Technology*: Models future break/tire wear and diesel-related pollution burdens and uses CalEnviroScreen to classify/disaggregate impacts across communities.
Health distribution mapping	Outcome is health, and an index is used to stratify or order geographic units to characterize inequalities in health burdens	Distributive justice (who bears health burdens)	[53–58]	Patel et al. (2023), *JAMA Internal Medicine*: Compares population health status across communities using the Environmental Justice Index.
Heterogeneity analyses	An index is used as an effect modifier in an exposure-outcome model	Distributive justice (health equity is understood through differential exposure-response functions; causal mechanisms can be ambiguous)	[59–62]	Niu et al. (2022), *JAMA Network Open*: Tests whether air-pollution–birthweight associations differ across neighborhood cumulative burden strata measured by CalEnviroScreen.
Policy targeting and equity evaluation	An index defines eligibility for a policy or is used to evaluate the distribution of benefits or impacts through scenario modeling	Distributive justice (who benefits); Procedural-by-proxy through rule design	[63–65]	Huynh et al. (2024), *Nature Machine Intelligence:* Audits CalEnviroScreen designation rules determine “disadvantaged community” status and funding allocation.
Data infrastructure	Indices are used as a source of data on component variables, with the complete component not used	Often not framed as justice; functions as a source of data on social determinants of health (environmental or otherwise)	[52, 55–59, 66–71]	Garland et al. (2025), *iScience*: Treats EPA EJScreen as a data source (environmental + demographic indicators) to build predictors.

The most common use is distributional mapping, where an index is used to stratify or order places and describe inequalities. Some studies focus on hazard distributions (who faces higher pollution concentrations, more hot days, or other environmental burdens), while others focus on health distributions (how morbidity or mortality burdens are patterned across higher- vs. lower-index communities). In these descriptive analyses, composite scores function as reduced-form measures of relative disadvantage. At the same time, many papers documenting disparities by race and socioeconomic status do not rely on screening tools directly; they operationalize distributive injustice using demographic indicators or custom constructs, sometimes assembled from variables drawn from the indices themselves [1, 72].

A second use case involves analyzing heterogeneity in exposure-health relationships. Here, indices enter epidemiologic models as covariates, stratification variables, or effect modifiers, serving as proxies for community-level vulnerability (social, economic, and structural conditions that influence a group’s risk of harm from environmental exposures and its capacity to prevent or respond to that harm) or susceptibility (differences in biological or health-related sensitivity that modify how a given level of environmental exposure affects individuals or populations). This approach links distributive justice to health equity by asking whether the same environmental exposure produces different health impacts in places with different levels of cumulative burden. But it can also compress multiple dimensions with distinct causal pathways, temporal dynamics, and policy meanings, into a single scalar measure, complicating interpretation of both effect heterogeneity and mechanism. Relatedly, some studies avoid using the full composite (or drop health components) to reduce collinearity or to avoid mechanically embedding outcomes into the model.

Because environmental justice indices are used in policy as administrative decision rules—to determine eligibility or priority for enforcement attention, permitting constraints, and public investment—scholarship has started to stress-test these tools against explicit equity objectives, rather than assuming targeting disadvantaged communities will reduce disparities. For example, Wang et al. (2023) [73] simulate emissions cuts targeted using the CEJST and show that this targeting can fail to reduce, and in some cases increase, racial/ethnic PM_2.5_ exposure disparities, because CEJST eligibility is not optimized for disparity minimization. Relatedly, Koolik et al. (2025) [40] argue that emissions reduction alone is not a disparity strategy: eliminating exposure gaps requires policies that directly target disparity metrics (and, in practice, the spatial distribution of sources and exposure determinants), not only average concentration declines.

A smaller body of work focuses on methodological critique and validation of indices, showing that technical design choices can materially change rankings, designations, and policy-relevant conclusions. Huynh et al. (2024) [63], for example, demonstrate that plausible specification changes to CalEnviroScreen can shift community eligibility and associated resource flows. Related evidence from climate adaptation allocation reaches a similar conclusion at a different scale: Pollack et al. (2025) [74] find that restricting investments to census-tract “Justice40 communities” can underperform simpler rules that target risk burden directly, highlighting the scale-mismatch risk embedded in tract-based designations. This scholarship foregrounds an epistemic justice concern: index design helps determine which definitions of environmental injustice become authoritative, and those definitions then carry real distributional consequences through policy and resource allocation.

## Discussion

This review shows that government-sponsored environmental justice indices are not neutral measurement tools but instead are data-scientific instruments that encode partial definitions of justice. Although these tools draw on environmental justice scholarship, their design emphasizes distributive burden in ways that make injustice legible to administrative systems while leaving other dimensions less visible. In this respect, environmental justice indices are one instance of a broader pattern: like poverty thresholds, credit scores, and criminal justice risk assessments, they convert complex social realities into administrative categories through choices that appear technical but carry high distributional stakes. In each case, the index — and the process of constructing it — risks defining the conditions it claims to measure. Despite their growing policy influence, however, the composite structure of these indices limits their interpretability for empirical research, perhaps helping explain their limited uptake as primary analytical tools in the peer-reviewed literature.

Across jurisdictions, environmental justice indices exhibit striking structural convergence. Most, modeled after CalEnviroScreen, rely on an exposure–vulnerability framework that summarizes environmental burdens and population characteristics through standardized indicators and combines them into composite scores. This convergence reflects both the availability of mappable data and the demand for metrics that can be applied consistently across policy contexts; however, it does not imply agreement on how justice should be defined. Because distributive burdens are more readily quantified and compared across places than procedural or recognitional dimensions, indices tend to frame justice as a characteristic of locations rather than as the outcome of political, historical, and institutional processes. This framing aligns well with funding allocation and regulatory screening, but less well with the broader goals of environmental justice scholarship, namely procedural and recognitional justice.

From a distributive perspective, the dominant exposure-vulnerability framework is consistent with quantitative characterizations of environmental justice. Both additive and multiplicative aggregation models aim to identify areas with high cumulative burden, but they encode different assumptions about how domains interact. Additive models treat exposure and vulnerability domains as contributing independently to the composite score, while multiplicative models emphasize their co-occurrence by amplifying scores where both are elevated. In both cases, justice is represented through relative rankings (typically percentiles or deciles), rather than through absolute exposure levels or health-based thresholds. This makes indices well-suited for within-geography comparisons and identifying the most burdened areas, but less suited for evaluating whether conditions exceed protective standards or for interpreting changes over time.

Procedural justice is not directly measured by these indices, but can nevertheless be shaped indirectly through the governance effects of their construction and use. Decisions about feature selection, scaling, weighting, aggregation logic, and thresholds influence how composite scores are interpreted and applied in regulatory, enforcement, and funding processes. When indices are used to define categorical designations, such as “disadvantaged communities,” modeling choices effectively determine eligibility for policy attention or resources. Importantly, variation in documentation quality and transparency additionally affects who can understand, reproduce, or challenge these tools.

Recognitional justice is the dimension least directly captured by current index designs. Composite indices inevitably abstract away local histories, lived experience, and community-defined priorities. While some tools include indicators intended to reflect historical disinvestment or structural disadvantage, these are incorporated into vulnerability domains rather than treated as distinct dimensions of environmental justice. As a result, communities are increasingly recognized primarily through their statistical resemblance to predefined categories of disadvantage, rather than through their own accounts of harm, resilience, or priorities.

Even when environmental justice indices are used to steer funding toward disadvantaged communities, such as by identifying priority areas through CEJST under the Justice40 Initiative, eligibility does not guarantee benefit. Among qualifying places, successfully obtaining and implementing funding often depends on administrative capacity: staff time, grant-writing skills, technical expertise, and institutional support [75–77]. These capacities are not evenly distributed and are frequently shaped by the same structural inequities the policies seek to address, making complex application and compliance requirements de facto barriers to access. Notably, programs supporting electric vehicles, rooftop solar, or building electrification are often taken up more readily by communities with greater organizational resources [64, 65]. In this way, factors not captured by indices, such as staffing, experience managing grants, and access to technical assistance, can shape who ultimately benefits, highlighting that distributive targeting alone does not fully address deeper procedural and structural barriers.

These limitations underscore the importance of treating environmental justice indices as partial representations, suited to some purposes but not others. Indices can make inequities visible at scale and support redistribution in contexts where resources are limited. But when they become the dominant, or sole, means by which justice is defined, they risk narrowing the scope of environmental justice to what can be computed, ranked, and thresholded. This, in turn, places a responsibility on both developers and end users to be transparent about what these tools do and do not capture. For index developers, this includes clearly articulating the intended purpose of the tool, the dimensions of justice it seeks to operationalize, and those it necessarily omits, as well as documenting how design choices, such as indicator selection, normalization, weighting, and aggregation, reflect these priorities. For researchers and other end users, transparency entails treating indices as partial and purpose-specific constructs rather than comprehensive measures of injustice, and explicitly stating how their use aligns with the justice dimensions under study. This requires a commitment to understanding how index structure shapes interpretation: which indicators are included or excluded; how variables are normalized, weighted, and aggregated; and what kinds of differences the final score can or cannot distinguish.

This risk is amplified by the institutional context in which these tools now operate. As environmental justice has moved into the administrative mainstream, screening indices and “disadvantaged community” designations are embedded in high-stakes policy decisions, shaping eligibility and funding priorities across major public programs. At the federal level, the Justice40 Initiative extended this framework across 518 covered programs, many relying on the CEJST for place-based targeting. The Inflation Reduction Act established large funding streams tied to benefits for disadvantaged communities, including EPA’s $2.8 billion Environmental and Climate Justice program and $200 million in technical assistance. In California, cap-and-trade revenues, totaling roughly $28 billion, flow through the Greenhouse Gas Reduction Fund, with SB 535 requiring at least 25% to benefit disadvantaged communities (and 10% to be invested within them), as identified by CalEnviroScreen.

At a moment when the assumptions encoded in these data-scientific tools carry increasing consequence, several of the indices reviewed here have undergone rapid revision, periods of unavailability, or loss of public documentation. Under these conditions, a critical understanding of how these indices are constructed is therefore not only a methodological concern, but a matter of democratic accountability. Index documentation should therefore be treated as a durable public record. At minimum, developers should maintain versioned documentation that explicitly records changes to indicator definitions, data sources, normalization procedures, weighting schemes, and aggregation methods across releases. Prior versions of both methods and scores should remain publicly accessible, with clear change logs that allow users to reconstruct how a community’s classification has shifted over time. Without this continuity, users cannot distinguish real changes in underlying conditions from changes induced by methodological revision, undermining transparency, reproducibility, and public trust.

Environmental justice indices sit within a broader ecosystem of place-based composite mapping tools that are often used in similar ways, but reflect different conceptual aims. In public health and emergency management, indices such as the Social Vulnerability Index and the Area Deprivation Index are routinely used to characterize community vulnerability and guide preparedness, targeting, or resource allocation. During the Covid-19 pandemic, the SVI was proposed as a tool to prioritize vaccine access [78], and the ADI has been embedded in Medicare payment models that adjust reimbursements to providers serving populations in areas with higher levels of deprivation [79, 80]. Hazard-focused tools such as FEMA’s National Risk Index map exposure and resilience to specific climate and disaster risks. FEMA has used the SVI in its Building Resilient Infrastructure and Communities grant scoring rubric, awarding priority points to applicants serving communities with SVI > 0.6 [81]. Beyond these general-purpose tools, many domain-specific composites have been developed to map vulnerability to particular climate hazards (e.g., heat or flooding) or to characterize pesticide-related risks in agricultural regions [82–84]. Although these instruments are not designed to operationalize environmental justice claims, they are frequently applied alongside (or substituted for) environmental justice screening tools in research and policy contexts.

Importantly, composite indices are shaped by many of the same forces that drive structural racism and historical disinvestment, but they rarely measure those processes directly. Without an explicit theory of what an index is intended to capture—and at what level (policy, institution, neighborhood, or individual)—it is easy to slide from interpreting an index as a snapshot of current conditions to treating it as evidence about upstream systems [85, 86]. One partial exception is CEJST, which includes an indicator intended to proxy historic underinvestment, but even there the mapping from past institutional practices to present-day vulnerability is indirect and incomplete.

## Conclusion

As environmental justice becomes increasingly encoded through data-scientific tools, it is essential to recognize that environmental justice indices are not neutral measurement devices but governance mechanisms that translate particular conceptions of justice into administrative practice. The design choices embedded in these indices—such as feature selection, normalization, weighting, aggregation logic, and thresholding—reflect institutional priorities, data availability, and policy needs, and they materially shape which communities are designated as burdened, disadvantaged, or eligible for intervention. These tools make distributive injustice legible at scale and are well-suited for regulatory screening and resource allocation, but they do so by privileging quantifiable exposures and standardized indicators over procedural, recognitional, and community-defined dimensions of justice.

Recognizing these limitations does not require abandoning environmental justice indices. Rather, it underscores the need to situate them within a broader ecosystem of environmental justice tools that includes community participation, qualitative evidence, historical analysis, and mechanisms for transparency and contestation. Without such context, the growing reliance on composite indices in high-stakes policy settings risks narrowing environmental justice to what can be computed, ranked, and thresholded, substituting technical classification for the more expansive justice claims that originally motivated the field.

## Key References

- Chowkwanyun M. Environmental Justice: Where It Has Been, and Where It Might Be Going. Annual Review of Public Health 2023; 44: 93–111.
  - ○ Offers a historically grounded, interdisciplinary review of environmental justice simultaneously as a social movement, scholarly field, and administrative project, highlighting coalition based “inside-outside” politics, mixed record in courts and regulatory agencies, and key directions for future scholarship and advocacy.
- Cain, Lucas; Hernández-Cortés, Danae; Timmins, Christopher; Weber, Paige (2024). “Recent Findings and Methodologies in Economics Research in Environmental Justice.” Review of Environmental Economics and Policy 18(1): 116–142.
  - ○ Provides an economics-oriented synthesis of recent environmental justice research, focusing on measurement choices and causal identification strategies shape inferences about disparities and the distributional impacts of environmental policy.
- Azzouz M, Hasan Z, Rahman MM, Gauderman WJ, Lorenzo M, Lurmann FW et al. Does socioeconomic and environmental burden affect vulnerability to extreme air pollution and heat? A case-crossover study of mortality in California. J Expo Sci Environ Epidemiol 2025; 35: 294–302.
  - ○ Uses a case-crossover design to test whether neighborhood socioeconomic and cumulative social burden (via CalEnviroScreen) modify acute mortality risks from extreme heat and air pollution in California.
- Wang Y, Apte JS, Hill JD, Ivey CE, Johnson D, Min E et al. Air quality policy should quantify effects on disparities. Science 2023; 381: 272–274.
  - ○ An analysis and policy discussion that engages with the design of air quality regulation to be designed and evaluated not only on average pollution reductions but also for changes in absolute and relative exposure disparities across racial/ethnic and socioeconomic groups.

## Supplementary Information

Below is the link to the electronic supplementary material.

Supplementary Material 1

## Data Availability

All of the data used in this study is publicly available.
