# Supplementary material for: Encoding Justice with Data: Environmental Justice Screening Tools and the Limits of Quantification
Source: Curr Environ Health Rep. 2026 May 30;13(1):24. doi: 10.1007/s40572-026-00543-9 (PMC13222189; doi:10.1007/s40572-026-00543-9)
Supplement: Supplementary file 1 — Supplementary Material 1 [file 40572_2026_543_MOESM1_ESM.docx]

**Supplementary Information**

**SI 1. List of search terms**

( ( "environmental justice" AND (index OR indices OR screening OR "screening tool" OR "composite index") AND (analysis OR evaluation OR model OR modeling OR regression OR "policy evaluation") ) OR ( "environmental justice screening" AND (EJSCREEN OR CalEnviroScreen OR CEJST OR "Environmental Justice Index" OR EJI) AND (health OR exposure OR disparit* OR mortality) ) OR ( ("Climate and Economic Justice Screening Tool" OR CEJST OR Justice40) AND (disparit* OR inequ* OR exposure OR "air pollution" OR PM2.5 OR emissions) AND (evaluate OR evaluation OR scenario OR model OR modeling) ) OR ( ("Climate and Economic Justice Screening Tool" OR CEJST) AND ("disadvantaged communities" OR DAC) AND (target* OR priorit* OR allocat* OR eligibility OR threshold*) ) OR ( (EJSCREEN OR "environmental justice screen*") AND (regression OR model OR stratified OR "effect modification" OR interaction OR "risk assessment" OR epidemiolog*) ) OR ( (CalEnviroScreen OR "CalEnviroScreen 3.0" OR "CalEnviroScreen 4.0") AND (health OR hospitalization OR mortality OR asthma OR birth OR "exposure-response") ) OR ( CalEnviroScreen AND (validate OR validation OR sensitivity OR weighting OR "principal component*" OR "factor analysis" OR robustness) ) OR ( ("Environmental Justice Index" OR EJI) AND (CDC OR ATSDR OR "Agency for Toxic Substances") AND (health OR mortality OR hospitalization OR disparit*) ) )
